# RE-IIBP Methylates H3K79 and Induces MEIS1-mediated Apoptosis via H2BK120 Ubiquitination by RNF20

**DOI:** 10.1038/srep12485

**Published:** 2015-07-24

**Authors:** Jin Woo Park, Kee-Beom Kim, Ji-Young Kim, Yun-Cheol Chae, Oh-Seok Jeong, Sang-Beom Seo

**Affiliations:** 1 Department of Life Science, College of Natural Sciences, Chung-Ang University, Seoul 156-756, Republic of Korea

## Abstract

Histone lysine methylation contributes to transcriptional regulation by serving as a platform for the recruitment of various cofactors. Intense studies have been conducted for elucidating the functional meaning of H3K79 methylation, and to date, the only known HMTase responsible for the modification was DOT1L. In this study, we report that the MMSET isoform RE-IIBP has HMTase activity for H3K79. It was uncovered that RE-IIBP up-regulates MEIS1 transcription through H3K79 methylation via recruitment to the *MEIS1* promoter. By means of proteomic and biochemical analysis, association of RE-IIBP with the E3 ubiquitin ligase RNF20 was demonstrated for synergistic activation of MEIS1 transcription via H3K79 HMTase activity. Furthermore, It was observed that RE-IIBP induces MEIS1-mediated apoptosis, which was dependent on H2BK120 ubiquitination by RNF20. These findings suggest RE-IIBP as another candidate for further studies to elucidate the mechanism of H3K79 methylation and its biological functions.

Chromatin remodeling, facilitated by various post-translational modifications to histones, is considered a critical factor in the transcriptional regulation of genes. Among histone modifications, lysine methylation, regulated by histone methyltransferases (HMTases) and demethylases, has a central role in the control of transcription. The yeast Dot1 and its human counterpart, DOT1L, are known to methylate lysine 79, located within the globular domain of histone H3. DOT1L binds to the phosphorylated CTD of RNAPII, and results in H3K79 methylation which is prerequisite to gene expression^1^. In DOT1L knockout mice, the deletion of DOT1L resulted in failure of hematopoietic homeostasis^2^. Crosstalk among histone modifications is involved in the control of various cellular processes and chromatin remodeling. Ring finger protein 20 (RNF20) is the major E3 ubiquitin ligase in mammalian cells, regulating chromatin through monoubiquitination of H2BK120 (H2BK120ub). As an important marker of transcriptional activation, H2BK120ub is prerequisite for histone H3K4 methylation and H3K79 methylation^3^,^4^. RNF20 functions as a putative tumor suppressor and positively activates p53 responsive gene by being recruited to the *mdm2* promoter through interaction with p53/TP53^5^. The DOT1L recognizes H2BK120ub as a docking site and methylates H3K79^3^,^4^. H3K4 methylation and H3K79 methylation are strongly associated in transcribed regions^6^, and crosstalk between them is highly related to H2B120ub in transcriptional activation.

Multiple myeloma (MM), a malignancy of mature plasma cells, has translocations involving the immunoglobulin heavy chain (IgH) locus on chromosome 14 in 70% to 80% of patients^7^,^8^. MMSET (WHSC1/NSD2) is linked to the IgH promoter in t(4;14)(p16;q32) translocation, and is found in 15% to 20% of multiple myeloma. Loss of the *MMSET* gene is considered to cause Wolf-Hirschhorn Syndrome^9^. The SET domain containing proteins, MMSET and isoform RE-IIBP, are known to have functions in transcriptional regulation, DNA repair, and RNA processing through diverse histone lysine methylation specificities toward H3K27, H3K36, and H4K20^10^,^11^,^12^,^13^,^14^,^15^,^16^. In particular, RE-IIBP is known for its H3K27 methylation activity, and represses *IL-5* gene transcription via HDAC interaction^10^.

On the other hand, PBX-related homeobox gene MEIS1 is up-regulated by RE-IIBP^17^. MEIS1 was first identified in murine myeloid leukemia cells as a viral integration site^18^. During embryogenesis, MEIS1 is involved in vascular development and hematopoiesis, and following birth, is present in the bone marrow^19^,^20^. MEIS1 acts as downstream of the MLL fusion protein to maintain a state of continuous proliferation and differentiation arrest, and has a crucial role in leukemogenesis^21^,^22^. The MEIS1 and PBX1 interaction motif is required to induce caspase-dependent apoptosis^23^.

In this study, we report that RE-IIBP has H3K79 HMTase activity. Proteomic analysis identified that RE-IIBP interacts with RNF20, and demonstrated that RE-IIBP-mediated H3K79 methylation was further increased by RNF20. Furthermore, H3K79 methylation by RE-IIBP was dependent on the RNF20-mediated ubiquitination of H2BK120. RE-IIBP was found to induce cellular apoptosis through transcriptional activation of MEIS1 via its H3K79 HMTase activity. Interaction with RNF20 further up-regulated the MEIS1-mediated apoptotic induction through RE-IIBP. These findings provide direct evidence for yet another H3K79 methyltransferase besides DOT1L, and suggest the existence of different mechanisms with diverse biological impacts mediated by the H3K79 methylation.

## Results

### RE-IIBP Has HMTase Activity for Histone H3 Lysine 79

Previous studies indicated that SET domain containing MMSET and isoform RE-IIBP may possess a number of different histone lysine specificities for methylation, including H3K4, H3K27, H3K36 and H4K20^10^,^11^,^12^,^13^,^14^. Given that dysregulation of MMSET proteins and subsequent alteration of the epigenetic modifications thereafter have been linked to several human diseases, we decided to further characterize the unidentified lysine specificity of RE-IIBP HMTase activity.

**Figure 1:**
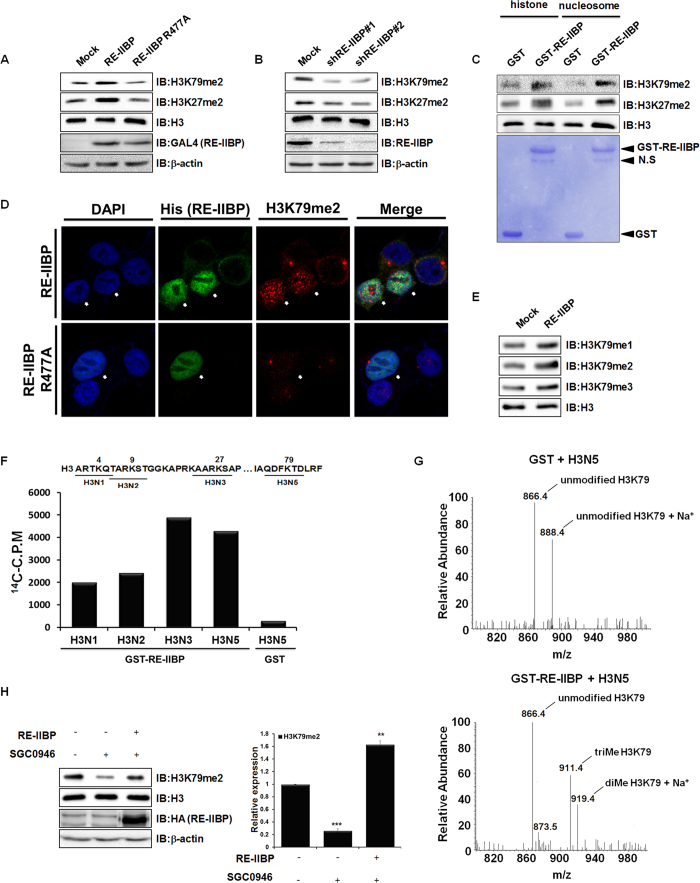
Histone H3K79 HMTase Activity of RE-IIBP. (**a**) and (**b**), H3K79me2 and H3K27me2 levels were analyzed in RE-IIBP or RE-IIBP R477A transfected 293T cells and stably knocked-down 293T cells using two independent shRNAs. Purified histones were immunoblotted with anti-H3K79me2, anti-H3K27me2, anti-RE-IIBP and anti-GAL4 antibodies. β-actin and H3 were used as loading controls. (**c**), Core histones and nucleosomes were used as substrates in the in virto HMTase assay with GST and GST-RE-IIBP. Incubated core histones and nucleosomes were subjected to Western blot. The amount of GST-RE-IIBP was determined by Coomassie staining. (**d**), 293T cells transiently transfected with His-RE-IIBP or His-RE-IIBP R477A were fixed, permeabilized, and immunostained with anti-His and anti-H3K79me2 antibodies. Nuclei were counterstained with DAPI. (**e**), Immunoblot analyses show the relative methylation levels of H3K79 in RE-IIBP transfected 293T cells. Purified histones using histone purification assay were immunoblotted with anti-H3K79me1, anti-H3K79me2, and anti-H3K79me3 antibodies. The methylation levels are normalized to H3. (**f**), HMTase assays were performed with four synthesized peptides, shown above, as substrates. Peptide methylations were measured using a scintillation counter. (**g**), Synthesized peptides (H3N5) were used as substrates in the HMTase assay with purified GST or GST-RE-IIBP. After 3 h, the peptides were analyzed by LC-MS. (**h**), SGC0946, DOT1L inhibitor, mediated H3K79me2 levels were restored by RE-IIBP. 293T cells treated with 1 nM SGC0946 were transiently transfected with RE-IIBP. Cells were lysed or used for histone purification assay. Each extracts were immunoblotted with anti-H3K79me2, anti-H3, anti-HA and anti-β-actin antibodies (left). The relative expression levels of H3K79me2 were quantified (right). Results are shown as means ± SDs n = 3; **p < 0.01, ***p < 0.001

In order to investigate the additional lysine specificity of RE-IIBP, the methylation levels of purified histones were screened after RE-IIBP transfection. H3K79 di-methylation (H3K79me2) was elevated, besides the previously identified H3K27me2 (Supplementary Figure S1A and Fig. 1A). When transfection with the RE-IIBP R477A, HMTase activity defective point mutant, was carried out, increase of the H3K79me2 levels did not occur (Fig. 1A). In contrast to H3K79 methylation, RE-IIBP did not change H3K9 and H3K4 methylation levels in our assay condition (Supplementary Figure S1A). RE-IIBP stably knocked-down 293T cell line was created by lenti-viral infection using two independent shRNAs, and the H3K79me2 levels were found to be significantly down-regulated (Fig. 1B). To further confirm the H3K79 HMTase activity of RE-IIBP, *in vitro* HMTase assays were performed using GST-RE-IIBP fusion protein and core histones or nucleosome, for which consistent results were obtained (Fig. 1C). RE-IIBP had similar HMTase activity toward core histones and necleosomes. In addition, confocal analysis was conducted using transiently RE-IIBP overexpressed 293T cells. This analysis also showed that RE-IIBP expression increased the H3K79me2 levels, confirming the H3K79 HMTase activity of RE-IIBP (Fig. 1D). However, in RE-IIBP R477A overexpressed cells, the change in H3K79me2 level was not detected (Fig. 1D). Both H3K79me2 images contain speckles showing similar patterns with γ-tubulin and they are speculated as non-histone proteins in centrosome detected non-specifically (data not shown). Upon examination of whether the RE-IIBP might mono-, di-, or tri-methylate H3K79, it was found that RE-IIBP can induce H3K79me1, H3K79me2, and H3K79me3 (Fig. 1E). Next, a more specific HMTase assay was conducted with histone H3 peptides, in which each six-amino-acid peptide contained one lysine residue. As a negative control, peptide H3N5 incubated with only GST protein was analyzed. Incubation with peptide H3N5 enhanced the strong methylation activity along with other peptides tested, which indicates that histone H3K79 is also a methylation target residue for RE-IIBP (Fig. 1F). To further confirm the lysine specificity of RE-IIBP, histone peptides were analyzed by LC-MS after the HMTase assay. The calculated molecular mass of the H3N5 peptide is 866 Da, for which each addition of a methyl group causes an increase of 14 to 15 Da. The nonmethylated H3N5 peptide had a main peak at 866.4 Da, while the di- and trimethylated peptides appeared at 919.4 and 911.4 Da, respectively, with the 23 Da mass of Na^+^ during the peptide sample preparation after the HMTase assay (Fig. 1G). The spectroscopy results clearly indicated that histone peptides H3N5 were di- and tri-methylated at H3K79. No peaks corresponding to the methylated forms of the H3N5 peptides were observed without the addition of RE-IIBP.

To prove that H3K79 methylation activity of RE-IIBP in the absence of DOT1L, 293T cells were treated with the SGC0946, DOT1L specific inhibitor, after which a significant decrease of H3K79me2 was observed (Fig. 1H). Consequently, when RE-IIBP was overexpressed in the SGC0946-treated cells, H3K79 methylation was successfully restored, proving the H3K79 HMTase activity of RE-IIBP (Fig. 1H). A DOT1L knocked-down stable cell line was created using lenti-viral infection, and overexpression of RE-IIBP successfully up-regulated the levels of H3K79me2 (Supplementary Figure S1B). Taken together, these results strongly indicate that RE-IIBP has H3K79 HMTase activity.

### H3K79 Methylation-Mediated Transcriptional Activation of MEIS1 by RE-IIBP

Numerous studies regarding the relationships between H3K79 methlyation and transcriptional regulation have been reported^24^. Therefore, we sought to screen the potential target genes of RE-IIBP. Selected genes were identified using real-time PCR. Transcription levels of *JMJD6*, *HSPA1A*, *WDR6*, and *MEIS1* were up-regulated by RE-IIBP overexpression and *USP36* level was not changed (Supplementary Figure S2). A member of the TALE family MEIS1 is a cofactor of HOX and increases HOX-DNA binding affinity. Increasing of HOX transcriptional activity by MEIS1 is known to contribute to accelerated development of leukemia. Besides, conjugation of MEIS1 with HOXA7 or HOXA9 has been found in various types of human cancer^25^,^26^. Similar to proto-oncogene c-Myc-mediated proliferation and apoptosis, overexpression of MEIS1 was found to induce apoptosis in various types of cells via interaction with cofactor PBX1^23^,^27^,^28^.

**Figure 2:**
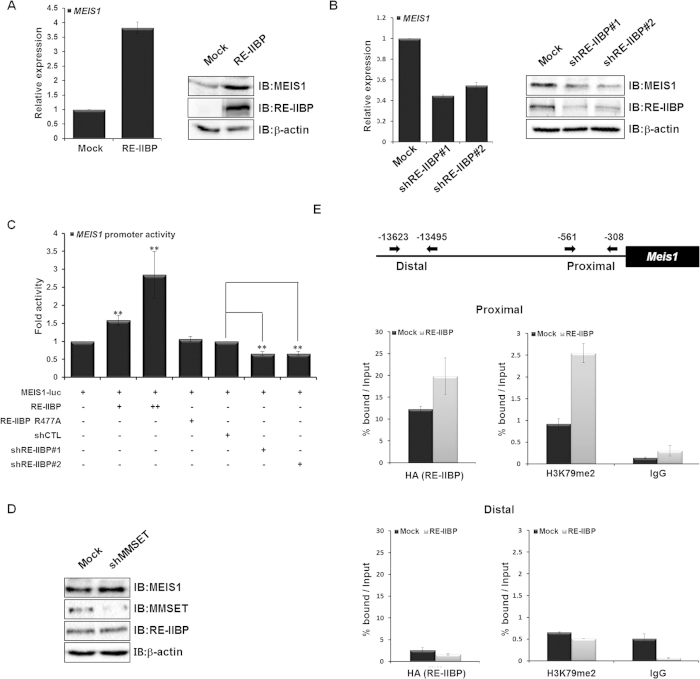
Transcriptional Activation of MEIS1 by RE-IIBP-Mediated H3K79 Methylation. (**a**) and (**b**), *MEIS1* mRNA levels were analyzed using real-time PCR in RE-IIBP transiently transfected 293T cells and stable RE-IIBP knocked-down 293T cells using two independent shRNAs (left). Cells were lysed and immunoblotted with anti-MEIS1 and anti-RE-IIBP antibodies. β-actin was used as a loading control (right). (**c**), 293T cells were transfected with pGL3-*MEIS1* promoter, increasing concentrations of GAL4-RE-IIBP constructs, GAL4-RE-IIBP R477A, shCTL, shRE-IIBP#1, and shRE-IIBP#2. Following transfection, cell extracts were assayed for luciferase activity. Luciferase activity was normalized to that of β-galactosidase. Results are shown as means ± SDs n = 3; **p < 0.01. (**d**), MMSET stably knocked-down K562 were analyzed by Western blot. (e), Schematic diagram of primer pairs in ChIP analysis (Top). 293T cells were transfected with HA-RE-IIBP. Following transfection, ChIP analysis was performed employing anti-IgG, anti-HA, and anti-H3K79me2 antibodies. The immunoprecipitated DNA fragments were analyzed by RT-PCR from the proximal (Top) and distal promoter regions of *MEIS1* (Bottom).

In addition, we previously reported that the RE-IIBP is up-regulated in the blood cells of various leukemic patients^10^. We supposed that RE-IIBP may regulate leukemia related gene expression. We previously screened RE-IIBP target genes in leukemia cell line and reported that the transcription of MEIS1 is regulated by RE-IIBP^17^. Extensive studies have been conducted for the role of MEIS1 in normal hematopoiesis and leukemia^22^. To further elucidate the detailed molecular mechanism of MEIS1 regulation by RE-IIBP, the effect of RE-IIBP overexpression on the levels of MEIS1 was first tested using real-time PCR and western blot in 293T cell lines. As expected, overexpression of RE-IIBP caused up-regulation of MEIS1 expression (Fig. 2A). In RE-IIBP stably knocked-down 293T cell line, MEIS1 expression was significantly down-regulated (Fig. 2B). In order to determine more precisely whether the transactivation of *MEIS1* by RE-IIBP is attributed to the H3K79 HMTase activity of RE-IIBP, a reporter assay was performed in 293T cells using a *MEIS1*-driven luciferase (luc) reporter system. Consistent with our other studies, *MEIS1* promoter activity was increased when RE-IIBP was transfected in a dose-dependent manner (Fig. 2C). Using the RE-IIBP R477A, the increase of *MEIS1* promoter activity by RE-IIBP was shown to be dependent on the HMTase activity, possibly through H3K79 methylation (Fig. 2C). As expected, decrease of *MEIS1* promoter activity was identified when RE-IIBP was knocked down using two different shRE-IIBP RNAs (Fig. 2C). Since RE-IIBP antibodies recognize both RE-IIBP and MMSET, a K562 stable cell line which only targets MMSET, but not RE-IIBP, was generated using shRNA against the *MMSET* HMG domain through lenti-viral infection (Supplementary Figure S3). Interestingly, knocked-down of MMSET did not affect MEIS1 expression (Fig. 2D).

To further elucidate the mechanism underlying the transcriptional regulation of *MEIS1* by H3K79 HMTase activity of RE-IIBP, a ChIP analysis with real-time PCR was performed. As expected, RE-IIBP recruitment and H3K79me2 levels at the *MEIS1* promoter were increased when RE-IIBP was overexpressed (Fig. 2E). Together, these findings suggest that RE-IIBP up-regulates *MEIS1* transcription through H3K79 methylation via recruitment to the *MEIS1* promoter.

### RE-IIBP Associates with RNF20 to Synergistically Activate *MEIS1* Transcription

**Figure 3:**
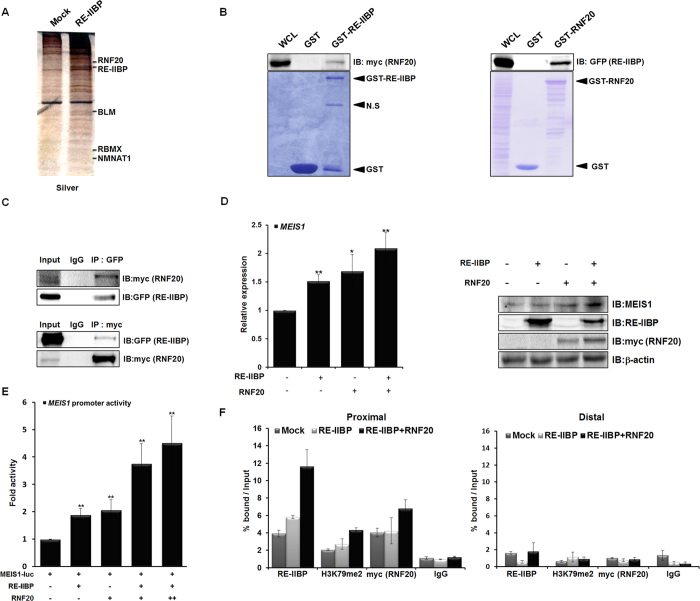
RNF20 Further Enhances Transcriptional Activation of MEIS1 by RE-IIBP. (**a**) Binding proteins to RE-IIBP were precipitated from cell extracts of 293T cells transfected with RE-IIBP. Differentially bound proteins were detected with silver staining and identified by LC-MS analysis. (**b**) Extracts from 293T cells transfected with myc-RNF20 or GFP-RE-IIBP were incubated with purified GST, GST-RE-IIBP or GST-RNF20. Associated proteins were eluted, resolved by SDS-PAGE, and immunoblotted by anti-myc and anti-GFP antibodies. The amount of RE-IIBP (left) and RNF20 (right) was determined by Coomassie staining. (**c**) Extracts from 293T cells transfected with GFP-RE-IIBP or myc-RNF20 were immunoprecipitated with anti-GFP, and anti-myc antibodies. Associated proteins were eluted, resolved by SDS-PAGE, and immunoblotted with anti-myc and anti-GFP antibodies. (**d**) *MEIS1* mRNA levels were analyzed using real-time PCR in RE-IIBP and RNF20 transfected 293T cells (left). Cells were lysed and immunoblotted with anti-MEIS1, anti-RE-IIBP and anti-myc antibodies (right). β-actin was used as a loading control. Results are shown as means ± SDs, n = 3; *p < 0.05, **p < 0.01. (**e**) 293T cells were transfected with pGL3-*MEIS1* promoter reporter and the indicated DNA constructs. Cell extracts were then assayed for luciferase activity. Luciferase activity was normalized to that of β-galactosidase. Results are shown as means ± SDs, n = 3; **p < 0.01. (**f**) DOT1L stably knocked-down 293T cells were transiently transfected with RE-IIBP and myc-RNF20. Following transfection, ChIP analysis was performed employing anti-IgG, anti-RE-IIBP, anti-H3K79me2, and anti-myc antibodies. The immunoprecipitated DNA fragments were analyzed by RT-PCR from the proximal (left) and distal promoter regions of *MEIS1* (right).

To gain a better understanding of the proteins that interact with RE-IIBP, GFP-RE-IIBP was overexpressed in 293T cells and GFP affinity purification was performed. The immunoprecipitated proteins were analyzed by liquid chromatography-mass spectrometry (LC-MS). Among the proteins found to interact with RE-IIBP, RNF20, a known E3 ubiquitin ligase, was observed (Fig. 3A).

To confirm the interaction between RE-IIBP and RNF20, the GST pull-down assay was first performed with purified GST-RE-IIBP and RNF20 overexpressed cell extracts (Fig. 3B). In addition, we confirmed this interaction by performing the experiment in reverse; that is, lysates from ectopically expressing RE-IIBP were incubated with GST-RNF20 (Fig. 3B). These experiments revealed noticeable interaction between RE-IIBP and RNF20. The interaction between the two proteins was confirmed *in vivo* via reciprocally conducted co-immunoprecipitation (IP) assays (Fig. 3C). It was next examined whether RNF20 affects the transcriptional regulation of *MEIS1* through RE-IIBP. It is interesting that the RE-IIBP-mediated up-regulation of MEIS1 expression was further activated, as shown by increasing expressions of MEIS1 in real-time PCR and western blot analysis (Fig. 3D). To further delineate the effects of RNF20 on RE-IIBP activity, a reporter assay was performed using a *MEIS1*-luc promoter. Consistent results were obtained in that RNF20 further increased the RE-IIBP-mediated transactivation of *MEIS1* (Fig. 3E). To investigate the role of the RE-IIBP/RNF20 complex at the *MEIS1* promoter, a ChIP analysis for the *MEIS1* promoter was performed in the DOT1L knocked-down stable cell line. Again, the recruitment of RE-IIBP to *MEIS1* promoter was higher when RNF20 was co-transfected (Fig. 3F). It was further identified that the H3K79 HMTase activity by RE-IIBP was also up-regulated in the presence of RNF20 (Fig. 3F). Together, these results suggest that RNF20 interacts with RE-IIBP and potentiates the RE-IIBP-mediated *MEIS1* transcription through increasing the H3K79 HMTase activity of RE-IIBP.

### Induction of MEIS1-Mediated Apoptosis by RE-IIBP is Dependent on H2BK120 Ubiquitination

**Figure 4:**
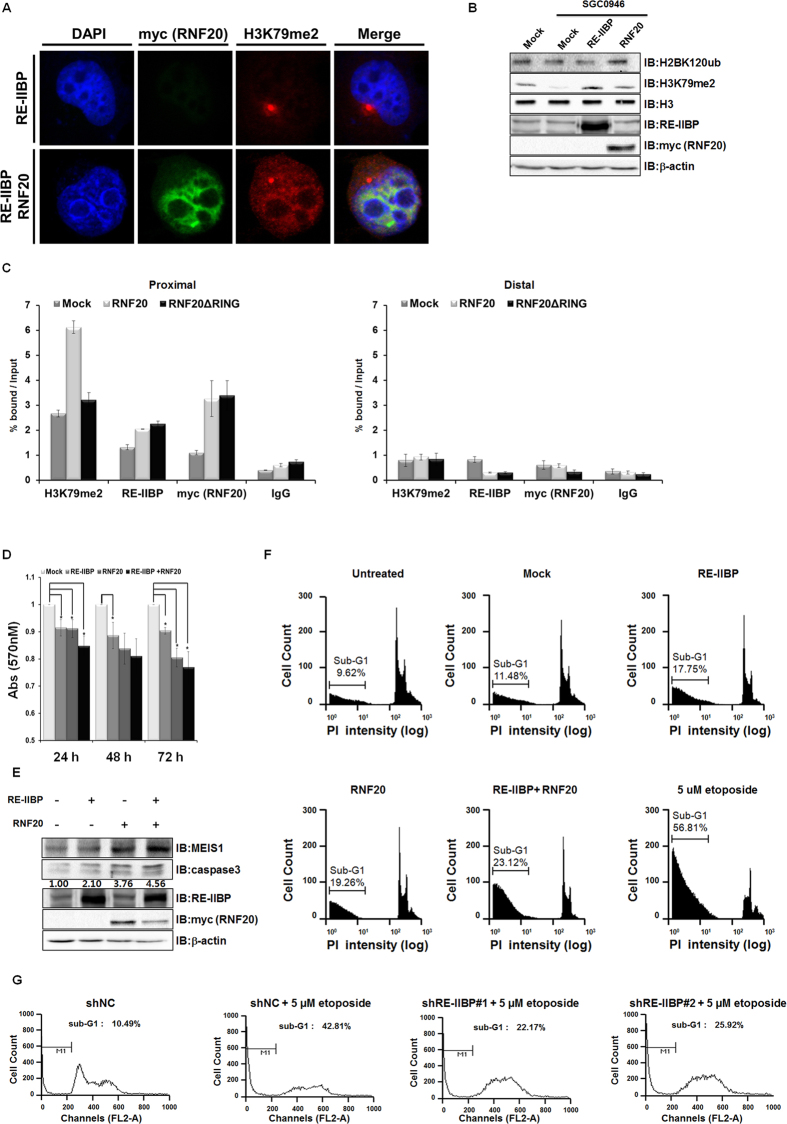
RE-IIBP Induces MEIS1-Mediated Apoptosis via H2BK120 Ubiquitination and H3K79 Methylation. (**a**) H1299 cells transiently transfected with RE-IIBP and RNF20 were fixed, permeabilized, and immunostained with anti-myc and anti-H3K79me2 antibodies. Nuclei were counterstained with DAPI. (**b**) 293T cells were transiently transfected with indicated plasmids and were treated with SGC0946 for 48 h. Cells were lysed or used for histone purification assay. Each extracts were immunoblotted with anti-H2BK120ub, anti-H3K79me2, anti-RE-IIBP and anti-myc antibodies. β-actin and H3 were used as loading controls. (**c**) 293T cells were transfected with RNF20 or RNF20 ΔRING. Following transfection, ChIP analysis was performed employing anti-IgG, anti-RE-IIBP, anti-myc (RNF20), and anti-H3K79me2 antibodies. The immunoprecipitated fragments were analyzed by RT-PCR form proximal (left) and distal promoter regions of *MEIS1* (right). (**d**) Cell viability was determined by the MTT assay. 293T cells were transfected with the indicated plasmids. Results are shown as means ± SDs, n = 3; *p < 0.05. (**e**) Immunoblot analyses show the relative expression levels of MEIS1 and caspase3 in RE-IIBP and RNF20 transfected 293T cells. The expression levels are normalized to β-actin. (**f**) Apoptosis of K562 cells transfected with the indicated plasmids was detected by PI staining. Cells were fixed, stained with PI for 30 min, and analyzed by FACS. (**g**) Apoptosis of K562 shRE-IIBP stable cells treated with etoposide was detected by PI staining. Cells were fixed, stained with PI for 30 min, and analyzed by FACS.

In a previous study, RNF20-mediated H2BK120ub was found to be prerequisite for H3K79 methylation^3^. Since RE-IIBP interacts with RNF20, we hypothesized that RNF20 might recruit RE-IIBP to target loci and increase the H2BK120ub-mediated H3K79 methylation. To investigate this possibility, confocal analysis was conducted using RE-IIBP and RNF20 co-transfected H1299 cells, we showed that overexpression of RE-IIBP up-regulated H3K79 methylation in Fig. 1D. In addition, RNF20 was co-localized with RE-IIBP in nucleus and further increased RE-IIBP-mediated H3K79 methylation levels (Fig. 4A and Supplementary Figure S4A). Consistent results were obtained that RNF20 further increased the RE-IIBP-mediated H3K79 methylation (Supplementary Figure S4B). To exclude the DOT1L-mediated H3K79 methylation, 293T cell line was treated with SGC0946, after which the H3K79 methylation levels were decreased (Fig. 4B). When RE-IIBP was overexpressed in the SGC0946-treated cells, the H3K79 methylation levels were restored (Fig. 4B). Intriguingly, overexpression of RNF20 increased not only the levels of H2bK120ub, but also that of H3K79 methylation (Fig. 4B). In addition, consistent results were confirmed using DOT1L knocked-down stable cell line (Supplementary Figure S4C). This strongly indicates that RNF20 increased RE-IIBP-mediated H3K79 methylation independent of H3K79 methylation by DOT1L. To determine whether RNF20 can regulate recruitment of RE-IIBP to the target gene, *MEIS1* promoter, a ChIP analysis was conducted with real-time PCR. The ChIP analysis demonstrated that the recruitment of RE-IIBP and the levels of H3K79me2 at the *MEIS1* promoter were increased when RNF20 was overexpressed (Fig. 4C). When RNF20ΔRING, RING domain deleted mutant, was overexpressed, levels of H3K79me2 were not changed. However, the recruitment of RE-IIBP remained increased in both RNF20 and RNF20ΔRING overexpression (Fig. 4C). In our preliminary data, RE-IIBP interacts with intermediate domain of RNF20 (Supplementary Figure S4D). This indicates that RNF20 interaction with RE-IIBP is RING domain independent. Still, RE-IIBP-mediated H3K79 methylation is dependent on H2BK120ub by RNF20. Taken together, these results indicate that RNF20 interacts with RE-IIBP and guides it to the target gene promoter for the enhancement of H2BK120ub-mediated H3K79 methylation by RE-IIBP.

Given that MEIS1 overexpression is known to induce apoptosis in a variety of cell types through a caspase-dependent process^23^, we next hypothesized that RE-IIBP might induce apoptosis through the activation of MEIS1 expression. To verify RE-IIBP induced apoptosis, RE-IIBP dependent cell survival rate was measured at indicated time points. The cell survival rate was decreased by RE-IIBP overexpression but increased by RE-IIBP knocked-down (Supplementary Figure S4E). To further confirm the effects of RE-IIBP on viability, the MTT assay was performed to measure cell survival at different time points, and consistent results were obtained as for the cell survival rate (Fig. 4D). Co-transfection of RNF20 and RE-IIBP further decreased for the cell survival rate (Fig. 4D).

Since RNF20 further enhanced the RE-IIBP-mediated induction of MEIS1, we next hypothesized that RNF20 might have an effect on RE-IIBP-mediated apoptosis. When RE-IIBP and RNF20 was overexpressed individually, increase of apoptosis marker caspase3 was detected. In addition, further up-regulation of the caspases3 was verified after co-transfection of RE-IIBP and RNF20, confirming that MEIS1-mediated apoptosis was induced by RE-IIBP and further enhanced by RNF20 (Fig. 4E). To further elucidate the biological role of RE-IIBP in cellular function, FACS analysis was performed to measure apoptosis. FACS analysis revealed that apoptosis was induced by the expression of RE-IIBP (sub-G1 6.27% increased) and RNF20 (sub-G1 7.78% increased), respectively. As a positive control, cells treated with etoposide were analyzed. Also, co-transfection of RE-IIBP and RNF20 resulted in a further increase in the number of cells entering apoptosis (sub-G1 11.64% increased) (Fig. 4F). Consistent results were obtained in FACS analysis using shRE-IIBP K562 stable cell, in which knocked-down of RE-IIBP inhibited etoposide-mediated apoptosis (24.64% or 16.89% decreased) (Fig. 4G). These results indicated that RE-IIBP induced MEIS1 expression via methylation of H3K79, and that interaction with RNF20 further enhanced RE-IIBP recruitment to the *MEIS1* promoter and activating MEIS1-mediated apoptosis.

## Discussion

In this study, we report that RE-IIBP has HMTase activity against H3K79. It was discovered that RE-IIBP induces apoptosis through transcriptional activation of *MEIS1* via its H3K79 HMTase activity. RE-IIBP interacts with RNF20, and H2BK120 ubiquitination by RNF20 further increases the RE-IIBP-mediated H3K79 methylation. Interaction with RNF20 increases the recruitment of RE-IIBP to the *MEIS1* promoter, suggesting that RNF20 has a synergistic effect on H3K79 methylation and the activation of target genes (Supplementary Figure S4F).

MMSET and its isoform RE-IIBP are known for their diverse lysine specificities towards H3K27, H3K36 and H4K20 methylation^10^,^11^,^14^,^15^. In addition, MMSET increases H3K4me3 *in vitro*, although this has not significantly been confirmed *in vivo*, indicating their wide repertoire of lysine specificities^29^. Recent study indicates that carboxyl terminal domain (CTD) of NSD family (NSD1, NSD2/MMSET/RE-IIBP, and NSD3/WHSC1L) can recognize and methylate H3K4, H3K9, H3K27, H3K36, H3K79, and H4K20 *in vitro*^30^. RNF20 targets H2BK120 monoubiquitination and stimulates the H3K79 methylation activity of DOT1L^31^,^32^. Similar histone crosstalk also applies to RE-IIBP-mediated H3K79 methylation and H2BK120 ubiquitination by RNF20. Using confocal and ChIP analysis, it was demonstrated herein that H2BK120 ubiquitination is prerequisite for H3K79 methylation by RE-IIBP, suggesting that the H3K79 methylation and H2BK120ub crosstalk might be universal.

We previously reported that RE-IIBP was overexpressed in leukemia cell lines and leukemia patient samples^10^. It is intriguing that screening of RE-IIBP target genes identified both up- and down-regulated genes. GATA1 and HOXA9 genes were repressed and MEIS1 gene was activated by RE-IIBP in leukemia cell line^17^. It is possible that RE-IIBP may play dual role in regulating transcription of target genes using H3K27 methylation and H3K79 methylation depends on specific condition. On the other hand, MLL fusion proteins recruit DOT1L to MLL target genes loci resulting in H3K79 methylation increase and leads to aberrant expression of a characteristic set of genes including HOXA9 and MEIS1 that drive leukemogenesis^33^,^34^

In this study, we report that RE-IIBP induces MEIS1-mediated apoptosis via H3K79 methylation and H2BK120 ubiquitination by RNF20 give synergistic effect to this process. It is possible that RE-IIBP maintains cells from undergoing transformation through the MEIS1-mediated apoptosis induction mechanism in normal stages but has other functions during abnormal stages, such as transformation. Dot1-mediated H3K79 methylation plays an important role in cell cycle regulation, DNA damage response, and transcription. Furthermore, mammalian DOT1L is an essential gene required for embryogenesis, hematopoiesis, and cardiac function^24^. In HeLa cells, there are high levels of H3K79me2 in G1 phase, which decrease in S phase, reach the lowest levels in G2 phase, and increase again in the M phase^35^. Our observations suggest that RE-IIBP, as another H3K79 HMTase, has a possible role in these biological pathways and in different cell cycles. The relationship between the only known H3K79 HMTase DOT1L and RE-IIBP in these processes will require more detailed investigation. Along the way, the identification of an H3K79 demethylase will further extend the understanding of the physiological role of H3K79 methylation.

## Methods

### Plasmid Constructs

The plasmids pCDNA3.1–RE-IIBP-His, pCDNA6-RE-IIBP-HA, GAL4-RE-IIBP, GAL4-RE-IIBP R477A, GFP-RE-IIBP and GST-RE-IIBP were previously described^10^. The plasmid pcDNA3.1-RNF20-myc-His was previously described^36^. RNF20, RNF20ΔRING (1-919 a.a), RNF20Δ2 (1-750 a.a), and RNF20Δ1 (1-300 a.a) coding sequences were subcloned into the bacterial expression vector pGEX-4T1 (Amersham Biosciences), thereby generating constructs for the production of glutathione S-transferase (GST)-tagged fusion proteins. RNF20ΔRING (1-919 a.a) coding sequences were subcloned into the mammalian expression vector pCDNA6 (Invitrogen). The *MEIS1* promoter region (−1345/+238) was amplified from human genomic DNA using primer pairs (Supplementary Table S1) and inserted into the KpnI/HindIII sites of the pGL3-basic vector (Promega). The short hairpin RNAs (shRNA) against human MMSET, RE-IIBP, DOT1L were designed using the siRNA sequence designer software (Clontech). A double-stranded oligonucleotide for shRNA plasmid construction was produced using primers from the 5' to the 3' end (Supplementary Table S1). These oligonucleotides were inserted into the AgeI/EcoRI site of the pLKO.1 TRC vector.

### Antibodies

Antibodies against H3K79me1 (ab2886), H3K79me2 (ab3594), H3K79me3 (Abcam; ab2621), H3K27me2 (07-452), caspase3 (04-439), H2BK120ub (17-650), H3K4me2 (07-030), H3K9me2 (Millipore ;07-441), NSD2/MMSET (Epicypher^TM^ ;13-0002), GAL4 (sc-577), β-actin (sc-47778), c-myc (sc-40), MEIS1/2 (sc-10599), GFP (sc-9996), H3 (sc-8654), His (sc-53073), and HA (Santa Cruz Biotechnology; sc-805) were employed.

### Cell Culture

293T cells were grown in Dulbecco’s modified Eagle’s medium (DMEM), and K562 and H1299 cells were grown in RPMI 1640 containing 10% heat inactivated fetal bovine serum and 0.05% penicillin-streptomycin at 37 °C in a 5% CO_2_ atmosphere. 293T, K562, and H1299 cells were transfected with the indicated DNA constructs using polyethylenimine (PEI) or Lipofectamine 2000 (Invitrogen). 293T cells were induced with 1 nM SGC0946^37^ and were harvested after 48 h.

### Histone Purification

Acid extraction of histones was performed as described in the literature^38^. Briefly, approximately 5 × 10^6^ cells were collected and washed once with PBS, then the cell pellet was resuspended in 1 ml of TEB lysis buffer (0.5% Triton X-100, 2 mM PMSF, and 1X protease inhibitor cocktail) and incubated for 30 min at 4 °C to promote hypotonic swelling. The cells were then recovered by centrifugation, resuspended in 400 μl of 0.2 M H_2_SO_4_, and incubated overnight on a rotator at 4 °C. After centrifugation, the supernatant was transferred and was treated with 132 ul 100% TCA for 30 min at 4 °C. The pellet was recovered by centrifugation, and washed with acetone, after which it was resuspended in deionized water.

### HMTase Assay

HMTase assays were carried out at 30 °C for 2 h in 30 ul volumes containing 50 mM Tris-HCl [pH 8.5], 20 mM KCl, 10 mM MgCl_2_, 10 mM beta-mercaptoethanol, 1.25 M sucrose, 100 μM cold-S-adenosyl-methionine (SAM) (Sigma) or 100 nCi of ^14^C-SAM (Perkin Elmer) and 1 μg core histones form calf thymus (Sigma) or nucleosome or histone peptides, and 2 μg of GST-RE-IIBP and GST. Peptides (H3N1, H3N2, H3N3, and H3N5) were synthesized based on the N-terminal amino acid sequences of H3 histone (Peptron). Proteins were separated via 15% sodium dodecyl sulfate-polyacrylamide gel electrophoresis (SDS-PAGE) and analyzed by immunobloting with the indicated antibodies. The peptides were filtered using p81 filter paper (Upstate) and washed three times with cold 10% trichloroacetic acid (TCA) and 95% ethanol for 5 min at room temperature. The filters were allowed to air dry, after which 2 ml of Ultima Gold (Perkin Elmer) was added, and the ^14^C-SAM was quantified using a scintillation counter. The HMTase assay, using cell lysates from RE-IIBP transiently transfected cells, was analyzed by immunobloting with the indicated antibodies.

### Immunocytochemistry

293T and H1299 cells were seeded in slides which were covered with collagen. Next day, cells were transfected with indicated plasmids. Cells were then washed with phosphate-buffered saline (PBS) and fixed with 4% paraformaldehyde and permeabilized with 0.2% triton X-100. After blocking with 1% BSA, cells were incubated with indicated antibodies, followed by incubation with FITC-conjugated anti-mouse, and Cy3-conjugated anti-rabbit antibodies (Jackson ImmunoResearch Laboratories). Finally, slides were mounted with GEL/MOUNT (Biomeda), and images were obtained by confocal laser scanning microscopy (Zeiss LSM 700).

### Immunoprecipitation and Mass Spectrometry

The 293T cells were transfected with GFP-RE-IIBP. After 48 h, cell lysates were immunopreicipitated with anti-GFP antibody. Protein A/G agarose beads were then added and rotated for 2 h at 4 °C. Bound proteins were resolved on SDS-PAGE, and visualized with silver staining. The eluted gel was subjected to LC-MS/MS sequencing and data analysis at the Korea Basic Science Institute. For the RNF20 interaction assay, the ectopically expressed GFP-RE-IIBP and myc-RNF20 cell lysates were immunoprecipitated with anti-GFP and anti-myc antibodies. Protein A/G agarose beads (GenDEPOT) were then added and rotated for 2 h at 4 °C. Bound proteins were analyzed by immunoblotting with anti-GFP and anti-myc antibodies.

### Luciferase Assay

Luciferase assays were conducted using a *MEIS1* promoter reporter system. 293T cells were co-transfected with the *MEIS1* promoter reporter construct and the indicated DNA constructs using PEI. Cells were harvested after 48 h and assayed for luciferase activity using a luciferase assay system (Promega). Each value was expressed as the mean of five replicates from a single assay. All experiments were performed at least three times.

### Reverse Transcription and Real-time PCR

Total RNA was isolated from transfected 293T cells using RNAiso Plus (TaKaRa). After cDNA synthesis, the cDNA was quantified and then subjected to analysis of mRNA expression. The PCR primers used are presented in Supplementary Table S1. Dissociation curves were examined after each PCR run to ensure amplification of a single product of the appropriate length. The mean threshold cycle (C_T_) and standard error values were calculated from individual C_T_ values obtained from triplicate reactions per stage. The normalized mean C_T_ value was estimated as ΔC_T_ by subtracting the mean C_T_ of β-actin. The value ΔΔC_T_ was calculated as the difference between the control ΔC_T_ and the values obtained for each sample. The n-fold change in gene expression, relative to an untreated control, was calculated as 2^–ΔΔCT^.

### Liquid Chromatography–Mass Spectrometry

Synthetic peptides (H3N5) (100 mM) were used as substrates in the HMTase assay with GST or GST-RE-IIBP. The reaction was stopped with 10% TCA precipitation for 10 min at 4 °C. After removing the precipitates by centrifugation for 10 min at 4000 rpm, the supernatants were retrieved and the methylated peptides were analyzed by liquid chromatography–mass spectrometry (LC–MS), carried out at the Korea Basic Science Institute. The eluted peptides were separated on a Luna column (C18 PepMap 100, 150 × 1 mm 5 micron) with a linear gradient (A: 5% ACN, 0.1% formic acid; B: 95% ACN, 0.1% formic acid) at a flow rate of 50 ml/min. Typically, 5 ml of sample was injected. Mass spectrometry was performed on a linear ion trap mass spectrometer (LCQ DECA XP, Thermo Finnigan) coupled to a nano-LC system (NANOSPACE SI-2, Shiseido). The MS scan range was 160–2000 m/z.

### Chromatin Immunoprecipitation Analysis

ChIP analysis was performed as described in the literature^10^. Briefly, 293T and the DOT1L knocked-down stable 293T cells were transfected with indicated plasmids. All cells were harvested after 48 h. The cells were cross-linked with the addition of 1% formaldehyde to the medium for 10 min at 37 °C, followed by the addition of 125 mM glycine for 5 min at room temperature. The cells were then lysed in SDS lysis buffer, and the samples were sonicated and immunoprecipitated using the indicated antibodies. The immunoprecipitates were eluted and reverse cross-linked. The DNA fragments were then purified and PCR amplified for quantification using each PCR primer pair (Supplementary Table S1). Disassociation curves were generated after each PCR run to ensure amplification of a single product of the appropriate length. The mean threshold cycle (C_T_) and standard error values were calculated from individual C_T_ values, obtained from duplicate reactions per stage. The normalized mean C_T_ value was estimated as ΔC_T_ by subtracting the mean C_T_ of the input from that of MEIS1.

### MTT (3-(4,5-dimethylthiazol-2-yl)-2,5-diphenyltetrazolium bromide) Assay

293T cells were seeded in 48-well plates (2.5 × 10^4^cells/well) and transiently transfected with RE-IIBP, RE-IIBP R477A, shRE-IIBP#1, shRE-IIBP#2, and RNF20. After 24, 48, and 72 h, MTT was added to the cells (200 μl, final concentration 0.5 mg/ml), after which they were incubated further for 4 h at 37 °C. The medium was then removed by aspiration, and DMSO was added (200 μl). The OD was determined using a spectrophotometer at the wavelength of 570 nm.

### FACS Analysis

To measure the effect of RE-IIBP on apoptosis, K562 shRE-IIBP stable cells treated 5 μM etoposide (SIGMA). Also, K562 cells were transfected with indicated plasmids using Lipofectamine 2000 (Invitrogen), and harvested 48 h later. Cells were trypsinized, washed, and fixed in ice-cold 70% ethanol for 30 min. Immediately before flow cytometric analysis, the cells were treated with RNase A (20 mg/ml) and stained with propidium iodide (SIGMA) for 30 min. The transfected cells were then subjected to FACS analysis using a BD Accuri C6 cytometer (BD Biosciences), and the data were analyzed using BD Accuri C6 software (BD Biosciences). The etoposide treated cells were subjected to FACS analysis using the FACSCalibur system (BD Biosciences).

## Additional Information

**How to cite this article**: Woo Park, J. *et al.* RE-IIBP Methylates H3K79 and Induces Meis1-mediated Apoptosis via H2BK120 Ubiquitination by RNF20. *Sci. Rep.* **5**, 12485; doi: 10.1038/srep12485 (2015).

## Supplementary Material

Supplementary Information
