# Supplementary material for: RE-IIBP Methylates H3K79 and Induces MEIS1-mediated Apoptosis via H2BK120 Ubiquitination by RNF20
Source: Sci Rep. 2015 Jul 24;5:12485. doi: 10.1038/srep12485 (PMC4513340; doi:10.1038/srep12485)
Supplement: Supplementary Information [file srep12485-s1.pdf]

Supplemental Information

RE-IIBP Methylates H3K79 and Induces MEIS1-mediated Apoptosis via H2BK120 Ubiquitination by RNF20

Jin Woo Park, Kee-Beom Kim, Ji-Young Kim, Yun-Cheol Chae, Oh-Seok Jeong, and Sang-Beom Seo

[1]Department of Life Science, College of Natural Sciences, Chung-Ang University, Seoul 156-756, Republic of Korea

[2]Present address: Division of Reproductive Science in Medicine, Department of OB/GYN, Feinberg School of Medicine, Northwestern University, Chicago, IL 60611, USA

***Correspondence: sangbs@cau.ac.kr**

Name	Sequence (5′- to -3′)	Purpose
pMEIS1(-1345/+238)-F	GGTACCCGCTGGAGAAAGAGCTAGT	promoter-luc
pMEIS1(-1345/+238)-R	AAGCTTGCTCCGTTTCCAAGACA	
shMMSET sense	CCGGGGGCATTGTTCAAGCAGAAGACTCGAGTCTTCTGCTTGAACAATGCCCTTTTTG	shRNA
shMMSET anti-sense	AATTCAAAAAGGGCATTGTTCAAGCAGAAGACTCGAGTCTTCTGCTTGAACAATGCCC	
shDOTL1 sense	CCGGCGCCAACACGAGTGTTATATTCTCGAGAATATAACACTCGTGTTGGCGTTTTTG	shRNA
shDOTL1 anti-sense	AATTCAAAAACGCCAACACGAGTGTTATATTCTCGAGAATATAACACTCGTGTTGGCG	
shREIIBP #1 sense	CCGGCGGAAAGCCAAGTTCACCTTTCTCGAGAAAGGTGAACTTGGCTTTCCGTTTTTG	shRNA
shREIIBP #1 anti-sense	AATTCAAAAACGGAAAGCCAAGTTCACCTTTCTCGGAAAGGTGAACTTGGCTTTCCG	
shREIIBP #2 sense	CCGGCCCAGAAAGAGCTTGGATATTCTCGAGAATATCCAAGCTCTTTCTGGGTTTTTG	shRNA
shREIIBP #2 anti-sense	AATTCAAAAACCCAGAAAGAGCTTGGATATTCTCGAGAATATCCAAGCTCTTTCTGGG	
MEIS1 promoter F	TTCAAGAGAGTCGGCTTTGG	ChIP
MEIS1 promoter R	GTCGCGTCTTACACAATGC	
MEIS1 disital F	TGAATGGTCCCCTAAGCACA	ChIP
MEIS1 disital R	AGGGAGCAGCAAGTGGTCTT	
MEIS1- F	CAAGGTGATGGCTTGGACAA	RT-PCR
MEIS1-R	CTCCGGGCATTAATAAACCAA	
DOT1L-F	CGCCCTGTCCCTGCACCTGC	RT-PCR
DOT1L-R	GCTCACTGGCAGCCGGCCTG	
HSPA1A-F	AGGACATCAGCCAGAACAAG	RT-PCR
HSPA1A-R	GTAGAAGTCGATGCCCTCAA	
SGK1-F	GGAAACTCAATCTGGGTGTG	RT-PCR
SGK1-R	CTGCTTCATGAAAGCTGGAT	
JMJD6-F	GAACTGGGATTCACATCGAC	RT-PCR
JMJD6-R	TCGGGTCACTTTGATGAGTT	
USP36-F	GCAGTGAGCACACGTATGAG	RT-PCR
USP36-R	ATTGAGAAAGCAGGTGTTGC	
WDR6-F	GGTCCACTCCTTTACACACG	RT-PCR
WDR6-R	CTAGCATGGTGGTGAGATCC	

Table S1. Primer pair in this study

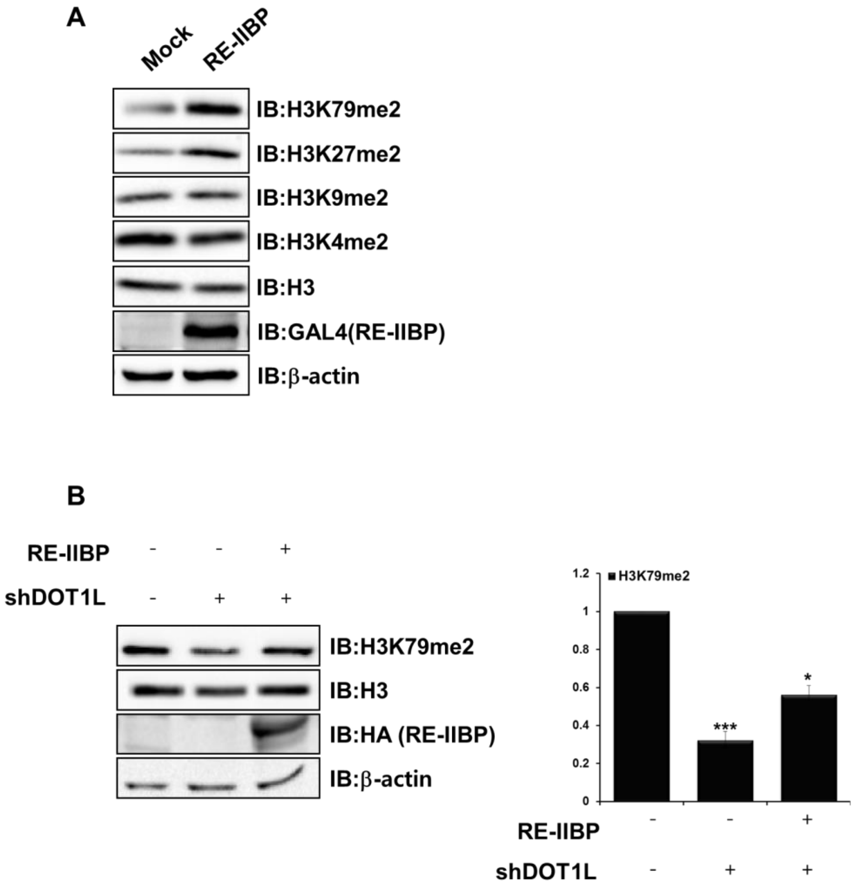

Figure S1. RE-IIBP has H3K79 methylation activity.

(a), HMTase activity was screened in RE-IIBP overexpressed 293T cells. Cells were lysed and immunoblotted with anti-H3K79me2, anti-H3K27me2, anti-H3K9me2, anti-H3K4me2 and anti-GAL4 antibodies. β-actin and H3 were used as loading controls. (b), Immunoblot analysis shows the relative methylation levels of H3K79 in DOT1L stably knocked-down and RE-IIBP rescued 293T cells. The methylation levels were normalized to H3 (left), and the relative expression level of H3K79me2 was quantified (right). Results are shown as means ± SDs n = 3; *p < 0.05, ***p < 0.001.

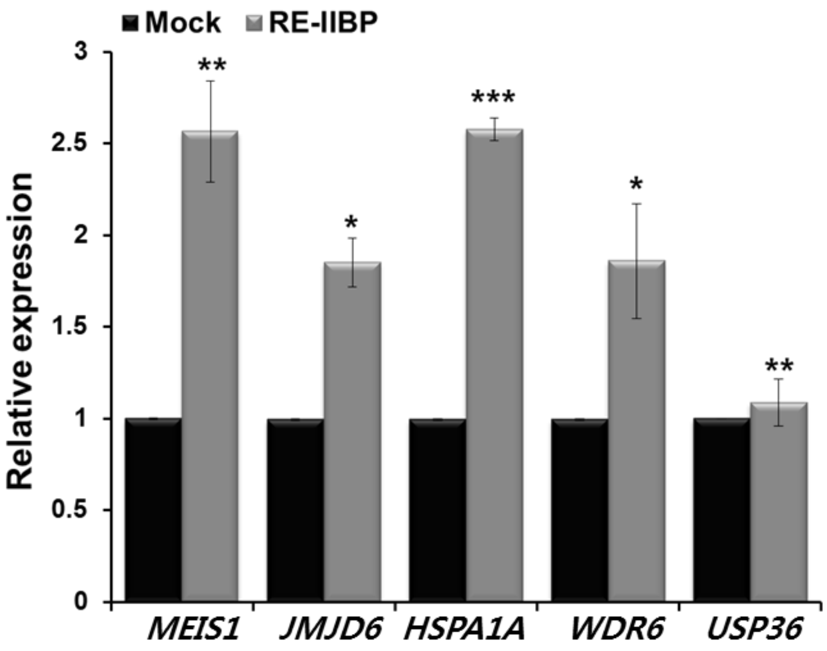

Figure S2. Screening of selected RE-IIBP target genes

(a), *MEIS1*, *JMJD6*, *HSPA1A*, *WDR6* and *USP36* mRNA levels were analyzed using real-time PCR in RE-IIBP overexpressed 293T cells. Each PCR primers used are presented in supplementary table S1. Results are shown as means ± SDs, n = 3; *$p < 0.05$, **$p < 0.01$, *** $p < 0.001$

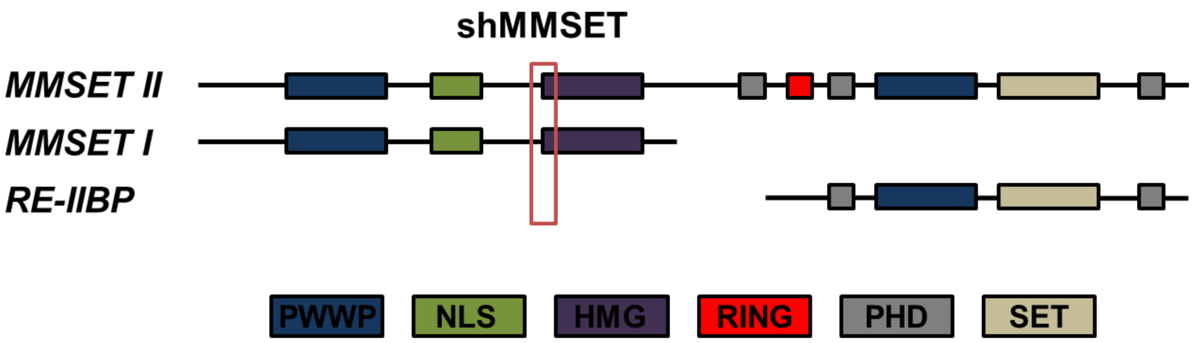

Figure S3. Protein domains of MMSET/WHSC1 isoform RE-IIBP

Schematic representation of *MMSETI*, *MMSETII*, and *RE-IIBP* domains. shMMSET RNA target regions are shown in the box.

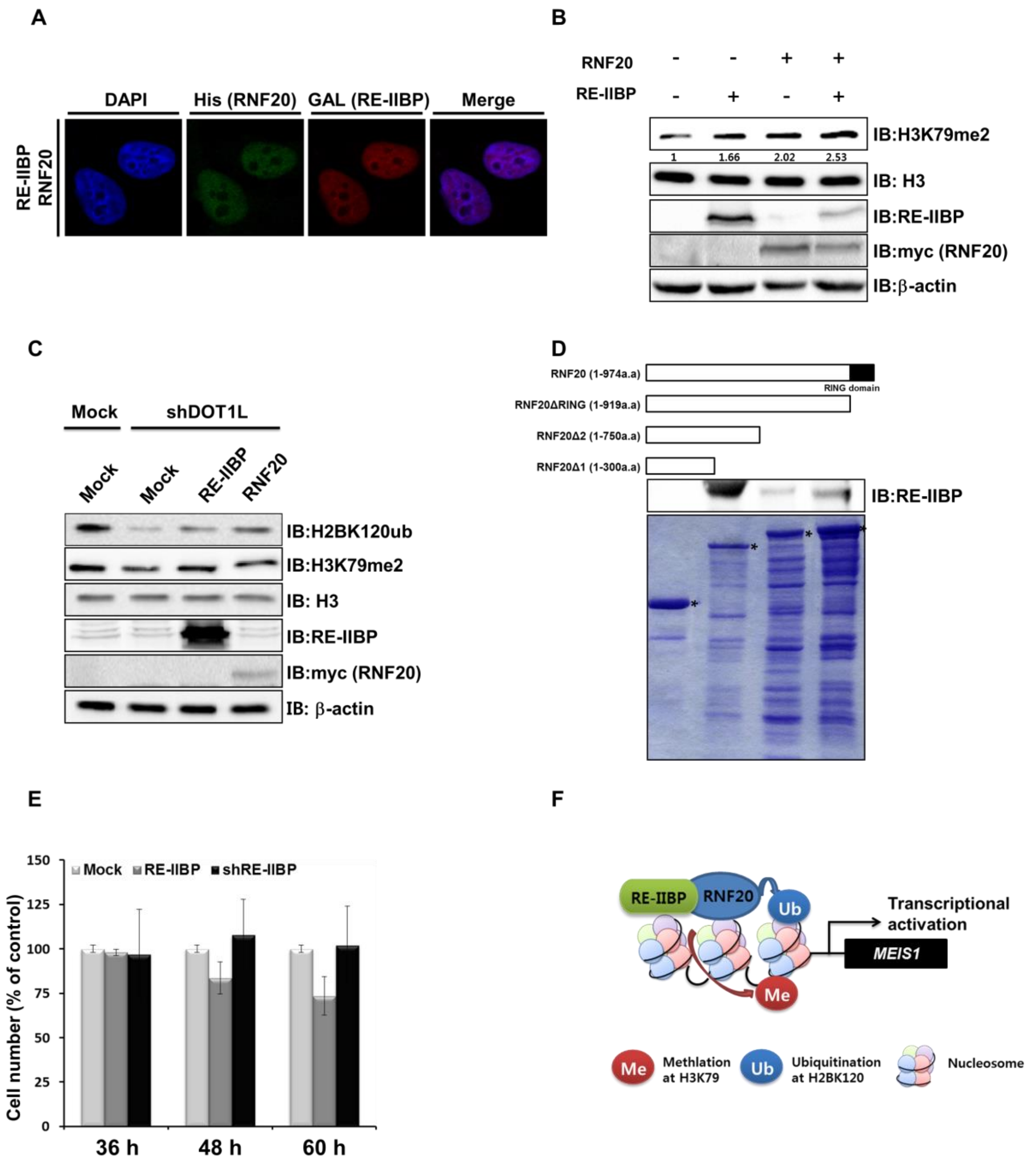
A
DAPI
His (RNF20)
GAL (RE-IIBP)
Merge
RE-IIBP
RNF20
B
RNF20 - - + +
RE-IIBP - + - +
IB:H3K79me2
1 1.66 2.02 2.53
IB: H3
IB:RE-IIBP
IB:myc (RNF20)
IB:β-actin
C
Mock
shDOT1L
Mock
Mock
RE-IIBP
RNF20
IB:H2BK120ub
IB:H3K79me2
IB: H3
IB:RE-IIBP
IB:myc (RNF20)
IB: β-actin
D
RNF20 (1-974a.a)
RING domain
RNF20ΔRING (1-919a.a)
RNF20Δ2 (1-750a.a)
RNF20Δ1 (1-300a.a)
IB:RE-IIBP
E
Mock
RE-IIBP
shRE-IIBP
Cell number (% of control)
150
125
100
75
50
25
0
36 h
48 h
60 h
F
RE-IIBP
RNF20
Ub
Transcriptional activation
MEIS1
Me
Me Methlation at H3K79
Ub Ubiquitination at H2BK120
Nucleosome

Figure S4

Figure S4. RE-IIBP-mediated H3K79 methylation is dependent on H2BK120 ubiquitination by RNF20

(a), 293T cells transiently transfected with RE-IIBP and RNF20 were fixed, permeabilized, and immunostained with anti-His and anti-GAL antibodies. Nuclei were counterstained with DAPI. (b), H3K79me2 levels were analyzed in RE-IIBP and RNF20 transfected 293T cells. Cells were lysed or used for histone purification assay. Each extracts were immunoblotted with anti-H3K79me2, anti-RE-IIBP and anti-myc (RNF20) antibodies. β-actin and H3 were used as a loading control. (c), DOT1L stably knocked-down 293T cells were transfected with RE-IIBP and RNF20. Cells were lysed or used for histone purification assay. Each extracts were immunoblotted with indicated antibody. (d), RE-IIBP overexpressed 293T lysates were incubated with GST-RNF20 and GST-RNF20 deletion constructs. Associated proteins were eluted, resolved by SDS-PAGE, and immunblotted with anti-RE-IIBP antibody. The amounts of full-length RNF20 and RNF20 deletion constructs were determined by Coomassie staining. (e), Cell survival was determined by the cell counting assay. Cells were transfected with RE-IIBP and shRE-IIBP for 36, 48, and 60 h, respectively. (f), Model for regulation of MEIS1 transcription through H3K79 methylation activity of RE-IIB

Figure S4
